# Towards the ictalurid catfish transcriptome: generation and analysis of 31,215 catfish ESTs

**DOI:** 10.1186/1471-2164-8-177

**Published:** 2007-06-18

**Authors:** Ping Li, Eric Peatman, Shaolin Wang, Jinian Feng, Chongbo He, Puttharat Baoprasertkul, Peng Xu, Huseyin Kucuktas, Samiran Nandi, Benjaporn Somridhivej, Jerry Serapion, Micah Simmons, Cemal Turan, Lei Liu, William Muir, Rex Dunham, Yolanda Brady, John Grizzle, Zhanjiang Liu

**Affiliations:** 1 The Fish Molecular Genetics and Biotechnology Laboratory, Department of Fisheries and Allied Aquacultures and Program of Cell and Molecular Biosciences, Aquatic Genomics Unit, Auburn University, Auburn, AL 36849, USA; 2 The W. M. Keck Center for Comparative and Functional Genomics, University of Illinois at Urbana-Champaign, Urbana, IL 61801, USA; 3 Department of Animal Sciences, Purdue University, West Lafayette, IN 47907, USA

## Abstract

**Background:**

EST sequencing is one of the most efficient means for gene discovery and molecular marker development, and can be additionally utilized in both comparative genome analysis and evaluation of gene duplications. While much progress has been made in catfish genomics, large-scale EST resources have been lacking. The objectives of this project were to construct primary cDNA libraries, to conduct initial EST sequencing to generate catfish EST resources, and to obtain baseline information about highly expressed genes in various catfish organs to provide a guide for the production of normalized and subtracted cDNA libraries for large-scale transcriptome analysis in catfish.

**Results:**

A total of 17 cDNA libraries were constructed including 12 from channel catfish (*Ictalurus punctatus*) and 5 from blue catfish (*I. furcatus*). A total of 31,215 ESTs, with average length of 778 bp, were generated including 20,451 from the channel catfish and 10,764 from blue catfish. Cluster analysis indicated that 73% of channel catfish and 67% of blue catfish ESTs were unique within the project. Over 53% and 50% of the channel catfish and blue catfish ESTs, respectively, had significant similarities to known genes. All ESTs have been deposited in GenBank. Evaluation of the catfish EST resources demonstrated their potential for molecular marker development, comparative genome analysis, and evaluation of ancient and recent gene duplications. Subtraction of abundantly expressed genes in a variety of catfish tissues, identified here, will allow the production of low-redundancy libraries for in-depth sequencing.

**Conclusion:**

The sequencing of 31,215 ESTs from channel catfish and blue catfish has significantly increased the EST resources in catfish. The EST resources should provide the potential for microarray development, polymorphic marker identification, mapping, and comparative genome analysis.

## Background

Catfish is the primary aquaculture species in the United States with an annual yield of over 600 million pounds [1]. While channel catfish (*Ictalurus punctatus*) accounts for the majority of commercial production, the closely related blue catfish (*I. furcatus*) possesses several economically important traits that have led to the production of an interspecific hybrid (channel female × blue male) recently available for commercial use [2,3]. Channel catfish is also an important model species for the study of comparative immunology, reproductive physiology, and toxicology. The channel catfish immune system is among the best characterized of any fish species, with decades of research leading to the establishment of clonal functionally distinct lymphocyte lines, panels of specific monoclonal antibody reagents for detection of catfish immunocytes, and characterization of much of the machinery of teleost adaptive immunity (see [4] for a summary).

Genome research requires the development of a number of resources that facilitate the organization of large amounts of genetic information into units that can be easily captured, mapped, and characterized. These resources include linkage maps, physical maps, bacterial artificial chromosome (BAC) libraries, and expressed sequence tags (ESTs). While BAC libraries and physical and linkage maps have been developed for catfish [5-11], large-scale EST resources have been lacking. Expressed sequence tag (EST) sequencing and analysis is an effective means for rapid gene discovery and annotation [12-19]. Large-scale EST projects have been carried out in several teleost species to date [20-22]. A successful EST project can quickly provide a wealth of genetic information for a species, often considerably shortening the laborious process of gene isolation. Large-scale EST projects provide the raw material for expression profiling experiments utilizing microarrays based on the transcript sequences. In addition to expression analysis, ESTs are vitally important to genome research in a given species. They provide a valuable source of gene-linked markers for linkage mapping [23], can be utilized in comparative genome analysis [24,25], and allow an assessment of gene duplications, a common phenomenon in teleost fish [26]. Sequencing the ESTs of two closely-related species such as channel catfish and blue catfish provides further benefits – gene identification is usually additive across the species, while molecular markers and gene orthologues are valuable for mapping and differentiating allelic and gene variants. Here we report the generation of 31,215 EST sequences from channel catfish and blue catfish and their potential for the development of molecular tools for mapping, genome analysis and expression profiling.

## Results and discussion

### cDNA library construction and sequencing of catfish ESTs

To obtain baseline information concerning the most abundantly expressed genes in catfish tissues and to capture a wide range of the transcriptome, we constructed cDNA libraries from various tissues of channel catfish and blue catfish (Table 1). These cDNA libraries were sequenced to generate the 31,215 ESTs reported here. Two of these libraries (channel catfish head kidney and spleen) have been previously reported [27,28], but were sequenced at greater depths in this project. Twelve of the cDNA libraries were produced from channel catfish tissues, and five from blue catfish tissues. Tissue libraries were produced by pooling tissue from fish experimentally infected with *Edwardsiella ictaluri *and tissue from healthy, control fish, to ensure that libraries included transcripts under both healthy and diseased conditions.

**Table 1:** A summary of cDNA libraries made from various catfish tissues and ESTs sequenced from these libraries. * indicates previously reported libraries used for additional sequencing in this project.

**Library**	**Species**	**Number of ESTs generated**
Gill	*I. punctatus*	2630
Head kidney*	*I. punctatus*	2614
Intestine	*I. punctatus*	2418
Liver	*I. punctatus*	1067
Muscle	*I. punctatus*	546
Olfactory	*I. punctatus*	390
Ovary	*I. punctatus*	1810
Pituitary	*I. punctatus*	1723
Spleen*	*I. punctatus*	3163
Stomach	*I. punctatus*	1369
Testes	*I. punctatus*	1377
Trunk kidney	*I. punctatus*	1344
		**Total = 20,451**
Head kidney	*I. furcatus*	2683
Heart	*I. furcatus*	659
Intestine	*I. furcatus*	1431
Liver	*I. furcatus*	4003
Spleen	*I. furcatus*	1988
		**Total = 10,764**

EST sequencing was conducted in two phases. In phase I, 200–300 clones were sequenced from each library to provide a list of the most abundantly expressed genes. In phase II, the most abundantly expressed genes (Supplemental Table 1) were subtracted from the clones to be sequenced by screening with overgo probes, to provide a higher gene discovery rate under a restricted budget. Overgo probes were designed for 200 genes, and the probes were used for colony lifting hybridization. Subsequently, only negative clones were picked for phase II sequencing. The number of ESTs generated from each library is given in Table 1. A total of 20,451 ESTs were successfully sequenced from channel catfish, and 10,764 ESTs were sequenced from blue catfish. These ESTs have been submitted to NCBI dbEST [GenBank: BM438128–BM439194, BQ096608–BQ097456, CF261473–CF266494, CF970744–CF972299, CK401558–CK426402, and EE993123–EE993655]. Furthermore, a database, ESTIMA: Catfish, was established for free public access [29]. These ESTs represent a significant fraction of the EST resources from channel catfish and the sole publicly available transcripts from blue catfish.

### Sequence assembly

A total of 31,215 clean sequences, with average length of 778 bp, were assembled using the CAP3 program [30] to evaluate the level of sequence redundancy. Blue catfish and channel catfish ESTs were assembled separately. Assembling of the 20,451 channel catfish sequences generated 1,848 clusters and 13,115 singletons. The average cluster contained 3.96 sequences. A total of 14,963 unique sequences were generated from channel catfish for this project. Assembling of the 10,764 blue catfish ESTs produced 881 clusters and 6,368 singletons with an average cluster size of 4.98 sequences. By this measure, sequencing of the blue catfish ESTs generated 7,249 unique sequences (Table 2). For the purpose of practical applications, we also performed clustering analysis by combining the ESTs from both channel catfish and blue catfish (data not presented here, but are available in the database). For instance, the clustering analysis of the ESTs from both species allowed design of microarrays with a larger set of unique sequences. For the identification of polymorphic microsatellites and SNPs, we also used ESTs from both species as our resource families were produced using the interspecific hybrids of channel catfish × blue catfish.

**Table 2:** A summary of clustering analysis and BLAST analysis of catfish ESTs.

	**Channel catfish (%)**	**Blue catfish (%)**
**Sequences**	20,451	10,764
**Contigs**	1,848	881
**Singletons**	13,115	6368
**Unique (%)**	14,963 (73%)	7249 (67%)
**Known (%)**	10,859 (53%)	5456 (50.7%)
**Unknown (%)**	9,592 (47%)	5308 (49.3%)

### Sequence annotation

The putative identities of the sequenced ESTs were determined using BLASTX searches against the non-redundant (*nr*) database in GenBank. Of the 20,451 channel catfish ESTs, 10,859 (53%) had significant hits (cutoff E-value of e^-5^), while the remaining 9,592 ESTs (47%) had no significant similarity to any sequences contained in GenBank (Table 2). Similarly, of the 10,764 blue catfish ESTs, 5,456 (50.7%) had significant hits (cutoff E-value of e^-5^), while the remaining 5,308 ESTs (49.3%) had no significant similarity to any sequences contained in the database. While a significant fraction of ESTs could not be identified by similarity searches, our results are comparable to other EST work in fishes. The unidentified transcripts are still valuable sources of microsatellite markers, and can be furthered sequenced if determined to be important in QTL analysis or expression profiling with microarrays. Additionally, many of these currently unknown transcripts will likely be identified when they cluster with additional transcripts produced in the future.

### Assessment of the sequenced catfish transcriptome

**Figure 1:**
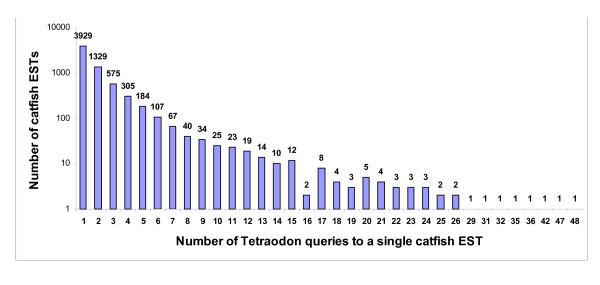
Bar graph of distribution of *Tetraodon *gene hits on catfish ESTs. For example, 575 catfish ESTs were each hit by three *Tetraodon *queries. A logarithmic scale was used for the Y-axis to better show the wide range of values.

To link these catfish EST resources to a comparative genome analysis framework, we conducted systematic TBLASTN searches on all existing catfish ESTs using *Tetraodon *chromosome-linked proteins as queries. The TBLASTN search parameters were set to select the top catfish hit and used a relatively more stringent cutoff E-value of e^-10^. Approximately 50% of annotated *Tetraodon *genes had a significant hit against catfish ESTs (Table 3), providing a rough assessment of the percentage of the catfish transcriptome now sequenced. However, BLAST-based comparisons between sequences of the two species have several shortcomings. First, rapid intraspecific diversification of gene families within catfish and *Tetraodon *has obscured gene homologies between the species. Second, short and/or divergent protein sequences would be excluded with the stringent parameters used. Altogether, 6,720 unique catfish ESTs were returned as the top hit of one or more *Tetraodon *proteins (Fig. 1). The factors mentioned above, especially gene family diversification, could also be responsible for the modest number of catfish hits. The majority of these catfish ESTs (3,929) were hit by a single *Tetraodon *query. However, a sizeable proportion (22%) were hit by three or more *Tetraodon *queries (Table 3, Fig. 1). A survey of the catfish ESTs hit by 20 or more *Tetraodon *queries revealed that these represented large gene families often functioning in developmental processes. Examples of the families hitting single catfish ESTs included the protocadherin clusters, notch proteins, zinc fingers, netrin family, and Hox proteins. Analysis of the chromosomal origins of these repetitive *Tetraodon *queries indicates that many are clustered tightly together and have likely resulted from rapid tandem gene duplication in their local environ [31,32]. High sequence conservation between members of these gene families may obscure their relationships with homologous families in catfish. Alternatively, transcripts representing gene family members in catfish may not have yet been sequenced.

**Table 3:** Summary of results of TBLASTN searches using all *Tetraodon *proteins as queries against catfish ESTs. A cutoff value of e^-10 ^was used, only the top catfish EST hit was selected.

Total number of *Tetraodon *protein queries	27,918
*Tetraodon *genes that had hit(s) to catfish ESTs	14,512
Number of catfish ESTs hit by a single *Tetraodon *gene	3,929
Number of catfish ESTs hit by a two *Tetraodon *genes	1,329
Number of catfish ESTs hit by a multiple *Tetraodon *genes	1,462

### Potential for comparative genome analysis and directed gene mapping

Comparative genome analysis is also an efficient approach for transferring linkage information from map-rich species to map-poor species [33]. In catfish, a gene-based genetic map is not yet available. In the absence of such a map, direct comparison of gene organization on chromosomes across species is difficult. However, the premise for comparative mapping is that many chromosome segments should be conserved among fish species. Of the 9,181 chromosome-linked *Tetraodon *proteins with significant hits on catfish ESTs, 2,529 hit a single EST. This subset should exclude many of the large intraspecific gene families and include genes with more apparent homologies. Concentrating on those ESTs with especially high *p*-values may further refine the set (Table 4). Associating the catfish ESTs with chromosome-linked *Tetraodon *proteins allows the development of a set of markers likely to be well-distributed across catfish chromosomes and which can provide anchors for a framework comparative map. Previously published analysis of microsatellite content of the ESTs described here, along with others in GenBank at the time of analysis, identified 4,855 microsatellites from 43,033 catfish ESTs. Of these, 4,103 were believed to represent unique genes [34]. Many of these microsatellites are being utilized for the construction of a gene-based linkage map for catfish. To make these markers more informative, the catfish ESTs hit by a single chromosome-linked *Tetraodon *query were searched for microsatellites. A total of 245 of these catfish ESTs contain a microsatellite and will aid in comparing the catfish linkage map to *Tetraodon nigroviridis*, providing an early assessment of genomic conservation between the two teleost species.

**Table 4:** BLAST results based on the chromosomal origin of the *Tetraodon *queries. Catfish ESTs hit by only a single *Tetraodon *gene were further parsed by alignment E-values. Undetermined *Tetraodon *genes are those whose chromosome location is not currently known.

**Chromosome**	**Number of Tetraodon genes with hit(s) to catfish ESTs**	**Number of catfish ESTs hit by a single Tetraodon gene**		
		
		**Total**	**With E-value of <e^-100^**	**With E-value of e^-50 ^to e^-100^**
1	798	231	18	84
2	809	209	21	75
3	623	179	17	76
4	263	74	10	22
5	351	98	15	38
6	225	59	7	16
7	503	129	17	46
8	404	114	13	32
9	458	114	15	44
10	496	142	12	42
11	544	143	20	62
12	503	149	6	51
13	484	141	18	44
14	372	117	19	45
15	584	147	13	40
16	390	99	15	28
17	360	86	13	33
18	454	137	18	53
19	171	37	1	15
20	75	25	4	9
21	314	99	11	31
Subtotal	9181	2529	283	886
Un	5331	1400	126	402
Total	14512	3929	409	1288

### Two species system for identification of ancient and recent gene duplications

**Figure 2:**
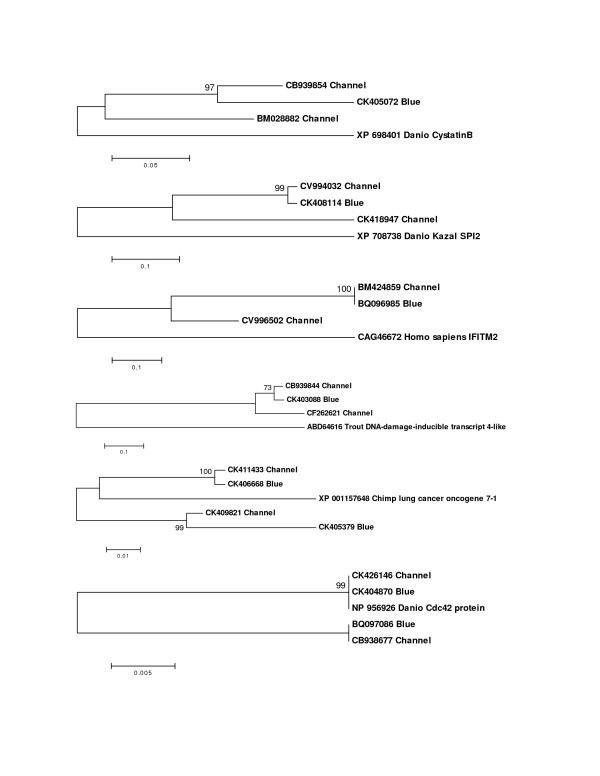
Selected examples of the ability to differentiate between catfish allelic variants and gene duplicates (paralogues) using both blue catfish and channel catfish sequences. Highly similar channel catfish sequences (Channel) and at least one blue catfish sequence (Blue) sharing the same BLAST identity were subjected to phylogenetic analysis. The topological stability of the neighbor joining trees was evaluated by 1000 bootstrapping replications, and the bootstrapping values are indicated by numbers at the nodes. Channel catfish and blue catfish genes placed into the same clade indicate that the additional, related channel catfish sequence is likely a paralogue rather than an allelic variant.

Gene duplication is a widespread phenomenon in vertebrate species and a particularly important trait of teleost fish. It has been proposed that a whole-genome duplication event occurred in the teleost lineage after its split from the tetrapod lineage, but that only a subset of the duplicated genes has been retained [35]. ESTs are a valuable tool for the identification of duplicated genes in species with and without a sequenced genome. For species with completed genome sequences, a large EST collection is invaluable for gene annotation in duplicated regions and facilitates the study of sub-functionalization among duplicated gene copies [36]. In species like catfish, where whole-genome sequencing is yet to be initiated, ESTs are important early indicators of gene copy numbers. However, comparisons of highly similar transcripts often do not allow researchers to differentiate between allelic variants and gene duplications. Using a two species system of EST sequencing and analysis, such as in channel catfish and blue catfish, can help to distinguish between these two possibilities. We surveyed the catfish EST resources for gene duplication events, applying the rationale that allelic variation within the same species should be smaller than the variation present between orthologues from different species [37]. Using this rationale, two highly similar channel catfish sequences would be considered paralogues if one of them is more closely related to a transcript from blue catfish than related to the other transcript from channel catfish. Likely instances of catfish gene duplication that included ESTs from both species were identified by BLASTN searches and then reciprocal BLASTX searches carried out. Most identified "duplications" were members of large, previously identified gene families that were the result of ancient gene duplications, i.e. similar members are present in other species such as *Danio rerio*. More informative were cases where all selected catfish ESTs were highly similar to the same BLASTX hit and/or gene copy number could not be predicted based on BLAST results, indicators of more recent gene duplication within catfish. Examples of these cases, where allelic variants could not be distinguished from gene paralogues based on the data from a single species alone, were subjected to phylogenetic analysis (Fig. 2). The putative blue catfish orthologues provided the context necessary to differentiate between the highly similar channel catfish transcripts. The ability to utilize the ESTs from the two closely related catfish species for analysis of local gene and genome-based duplications was one of the reasons for continuing EST sequencing efforts in catfish conducted by the Joint Genome Institute (see below).

### Subtraction probes for normalization of cDNA libraries

Sequencing a large number of cDNA libraries widened the range of the catfish transcriptome sequenced while providing information concerning the most abundantly expressed genes in a variety of tissue types. This information is critical to ensure high numbers of unique transcripts can be obtained when sequencing a library to greater depths. To produce a list of most abundantly expressed genes for further subtraction, we conducted cluster analysis of all catfish ESTs in the dbEST database of NCBI. Clusters were sorted by size and those containing 2 or more transcripts per 10,000 sequences were selected as subtraction drivers for use during the construction of normalized/subtracted cDNA libraries to be used for a large-scale EST project (Supplemental Table 2). Through the Community Sequencing Program, a project for sequencing 300,000 clones of catfish ESTs was recently approved by the Joint Genome Institute (JGI) of the Department of Energy (DOE). Subtraction of highly abundant genes based on information gained through the current project should markedly increase the number of unique transcripts obtained by JGI sequencing, and initial sequencing and quality control determination by JGI of the subtracted cDNA libraries we produced using this strategy confirmed this assessment.

## Conclusion

A large number of cDNA libraries have been made from both channel catfish and blue catfish, and they should be valuable resource for various molecular studies and for the construction of normalized cDNA libraries. This work is the first large-scale EST project in catfish. In addition to significant expansion of the channel catfish EST resources, and generation of the sole source of the blue catfish EST resource, the sequencing of 31,215 ESTs from channel catfish and blue catfish has provided the potential for the development of a number of molecular tools valuable for genome research. The EST resources will be particularly useful as sources of polymorphic markers including microsatellites and single nucleotide polymorphisms (SNPs) for gene mapping. In addition, the EST resources have aided in the identification and characterization of important genes involved in immune response [38,39]. The generated sequences are currently being utilized as reference points in comparative genome analysis and have been validated as an important tool for the assessment of gene duplications in catfish. Additionally, the ESTs served as a foundation for the creation of normalized, subtracted cDNA libraries currently being used for the sequencing of 300,000 ESTs from both ends by JGI. The development of microarrays [40,41] and linkage maps based on the catfish EST resources will further extend their applications in research.

## Methods

### Tissue samples and RNA isolation

All procedures involving the handling and treatment of fish used during this study were approved by the Auburn University Institutional Animal Care and Use Committee (AU-IACUC) prior to initiation. Channel and blue catfish were raised in troughs in the hatchery of the Auburn University Fish Genetics Hatchery for four weeks before harvesting of tissues. To create resource cDNA libraries containing a full complement of gene transcripts, including those expressed after infection, both healthy and infected catfish were used. Channel catfish and blue catfish were challenged with *Edwardsiella ictaluri *using procedures adapted from Dunham et al. [42]. Fish were divided into 2 groups, the non-challenged controls and the fish for challenge (N = 240). The fingerlings used for disease challenge were placed into a 150 L tank containing 1.1 × 10^6 ^*E. ictaluri *cells/ml for 1 h. The challenged fish were then removed and stocked into a 1000 L tank. At time of sampling, fish were euthanized with MS-222 at 300 mg/L before dissection. Tissue samples were collected from 15 control and 5 infected fish each at 24 h, 3 d, and 7 d during the challenge, pooled, quick-frozen in liquid nitrogen, and stored at -80°C until RNA extraction. The following tissues were collected: channel catfish gill, head kidney, trunk kidney, intestine, liver, skeletal muscle myomere, olfactory organ, ovary, pituitary, spleen, stomach, and testes; blue catfish head kidney, heart, intestine, liver and spleen. Equal tissue weights of all the control and infected pools for each tissue within a species were combined, ground to a fine powder with mortar and pestle in the presence of liquid nitrogen and thoroughly mixed. A fraction of the tissue samples was used for RNA isolation. Total RNA was isolated following the guanidium thiocyanate method [43] using the Trizol reagent (Invitrogen, Carlsbad, CA) following manufacturer's instructions. Poly(A)^+ ^RNA was purified from total cellular RNA using the Poly(A)^+ ^Pure kit (Ambion, Austin, TX) according to the manufacturer's instructions.

### Library construction

Initial sequencing of four catfish cDNA libraries, channel catfish brain, head kidney, skin, and spleen, was previously reported [27,28,44,45]. Fifteen additional libraries from the tissues listed above were constructed here closely following protocols used previously. Briefly, the cDNA libraries were constructed using the pSPORT-1 Superscript Plasmid Cloning System from Invitrogen. This cloning system provides a vector with capacity for uni-directional cloning of cDNAs that support choices of EST sequencing from either the 5'-, or 3'-end of the transcript. In this work, all ESTs were sequenced from upstream of the transcripts (5' sequencing) to provide a longer length of ESTs. Two micrograms of Poly(A)+ RNA were used in each initial reaction. Procedures followed instructions provided by the manufacturer with the exception that ElectroMax DH12S cells (Invitrogen) were used for electroporation of the cDNA library. The quality of the cDNA libraries was determined by number of primary recombinants and average insert size. Before sequencing analysis, the primary cDNA libraries were amplified once [46]. The pooled libraries were frozen in liquid nitrogen and stored at -80°C.

### Colony lifting hybridization and sequencing

Colony-lifting hybridization [46] was conducted using overgos as probes to reduce the sequencing redundancy of a set of 200 genes determined to be highly expressed by preliminary sequencing of the libraries. Oligonucleotides were custom made by Sigma Genosys (St. Louis, MO). Overgos were designed to overlap for 8 bases where the sense and antisense oligos pair, leaving the remaining 5' overhang for filling in using labeled nucleotides, P^32^-dATP and P^32^-dCTP [47,48]. All the overgos were labeled in a single reaction. Overgo hybridization was conducted at 45°C overnight using conditions as previously reported [49]. The filters were washed using 2× SSC at room temperature four times for 15 minutes each. After exposure of X-ray films, bacterial plates were aligned to match the patterns of the exposed colonies on the X-ray film. Negative colonies were picked for sequencing and manually arrayed into 384 well plates containing LB with antibiotics and 10% glycerin and stored at -80°C until sequencing. Sequencing was conducted using ABI PRISM 3730 automated sequencers located in the Core Facility of Purdue University.

### Sequence analysis, EST clustering, and sequence annotation

ESTs were trimmed for vector and adaptor sequences. Base calling was performed using the Phred program with quality cut-off set at 20. Sequences were assembled in CAP3 using a criteria of a minimum overlap of 70 bp sharing 90% sequence identities for clustering. Cleaned ESTs were used as queries for BLASTX searches against the *nr *database at NCBI and annotated based on the top, informative BLAST hit. A cutoff E-value of e^-5 ^was used for annotation. The channel catfish and blue catfish ESTs were submitted to dbEST. A database was developed to facilitate information dissemination. ESTs were annotated using the Gene ontology (GO) terms and the results built into the database.

### Catfish ESTs and comparative analysis

Chromosome-assigned proteins of *Tetraodon nigroviridis *as well as those from undetermined chromosome locations were downloaded from the protein database of NCBI. All proteins linked to a given *Tetraodon *chromosome were uploaded separately as query files onto the University of Illinois Keck Center's Gridblast server. All catfish ESTs from NCBI's dbEST were uploaded as a database on the same server. The TBLASTN search parameters were set to select the top catfish hit, using a cutoff E-value of e^-10^. Resulting text files were parsed to obtain *Tetraodon *query IDs, catfish hit IDs, and e-values and these were imported into Excel spreadsheets. Results were further sorted to separate those catfish ESTs hit by a single *Tetraodon *query and those hit by multiple *Tetraodon *queries. Catfish ESTs hit by a single *Tetraodon *query were uploaded to Msatfinder [50] to search for microsatellites contained in the sequences. BLASTX searches were carried out on those catfish ESTs hit by 20 or more *Tetraodon *queries.

### Assessment of gene duplication

Channel catfish TIGR consensus (TC) sequences, composed in part by the ESTs reported here [51] were used as queries for BLASTN searches against the *est_others *database of NCBI, limiting the entrez query to *Ictalurus*. Top hits with perfect matches (E-value = 0.0) were the channel catfish sequences from the TC. If additional highly similar hits (E-value <e^-25^) from both channel catfish and blue catfish ESTs were present, these sequences were noted for further analysis as potential gene duplicates. Reciprocal BLASTX searches were carried out using at least three ESTs from the initial searches, with at least one of these ESTs from blue catfish. When all ESTs shared the same top BLASTX hit, they were translated, and areas of amino acid overlap identified. Phylogenetic trees were drawn by the neighbor-joining method [52] within the Molecular Evolutionary Genetics Analysis (MEGA 3.0) package [53]. Data were analyzed using Poisson correction and gaps were removed by complete deletion. The topological stability of the trees was evaluated by 1,000 bootstrapping replications.

## Authors' contributions

PL constructed the cDNA libraries and participated in the sequencing efforts; EP conducted bioinformatic analysis and drafted the manuscript; SW and LL conducted bioinformatic analysis and constructed the database; JF, CH, PB, PX, HK, SN, BS, JS, MS, and CT participated in the sequencing and subtraction efforts; WM, RD, YB, and JG served as co-P.I.'s of the project for the overall project design, and ZL served as the P.I. for the overall design and execution of the project, and manuscript preparation. All authors read and approved the final manuscript.

## Supplementary Material

Additional file 1 The most abundantly expressed genes in various tissues of catfish. This file contains two Tables with Supplemental Table 1: Abundantly expressed transcripts in catfish cDNA libraries as determined by preliminary sequencing. Overgo probes were designed based on the given clone and used for subtraction of clones picked for further sequencing, and Supplemental Table 2: Abundantly expressed transcripts (>2 copies/10,000 transcripts) in catfish EST collection in NCBI's dbEST following the current project. Approximately 40,000 catfish ESTs were assembled. Indicated clones were used as drivers in subtraction of normalized cDNA libraries currently being sequenced by JGI. Click here for file
