# Supplementary material for: Towards the ictalurid catfish transcriptome: generation and analysis of 31,215 catfish ESTs
Source: BMC Genomics. 2007 Jun 18;8:177. doi: 10.1186/1471-2164-8-177 (PMC1906771; doi:10.1186/1471-2164-8-177)
Supplement: Additional file 1 — The most abundantly expressed genes in various tissues of catfish. This file contains two Tables with Supplemental Table 1: Abundantly expressed transcripts in catfish cDNA libraries as determined by preliminary sequencing. Overgo probes were designed based on the given clone and used for subtraction of clones picked for further sequencing, and Supplemental Table 2: Abundantly expressed transcripts (>2 copies/10,000 transcripts) in catfish EST collection in NCBI's dbEST following the current project. Approximately 40,000 catfish ESTs were assembled. Indicated clones were used as drivers in subtraction of normalized cDNA libraries currently being sequenced by JGI. [file 1471-2164-8-177-S1.doc]

**Supplemental Table 1: Abundantly expressed transcripts in catfish cDNA libraries as determined by preliminary sequencing. Overgo probes were designed based on the given clone and used for subtraction of clones picked for further sequencing.**

| **Clone Name** | **Putative Id** |
| --- | --- |
| IpBrn00018 | Immunoglobulin gamma heavy-chain |
| IpBrn00061 | Ribosomal protein L39 mrna, complete cds |
| IpBrn00066 | Trna-Val |
| IpBrn00086 | 40S ribosomal protein S15a mrna, complete cds |
| IpBrn00108 | Cytochrome c oxidase I |
| IpBrn00121 | Calmodulin |
| IpBrn00202 | 40S ribosomal protein S23 mrna, complete cds |
| IpBrn00221 | Ependymin (cold acclimation-related) |
| IpBrn00301 | 12S rrna gene |
| IpBrn00853 | Beta-5 tubulin |
| IpBrn00891 | Ribosomal protein L23a mrna, partial cds |
| IpBrn00892 | 40S ribosomal protein S9 mrna, complete cds |
| IpBrn01069 | 40S ribosomal protein S30 mrna, complete cds |
| IpBrn01136 | 40S ribosomal protein S19 mrna, complete cds |
| IpBrn01374 | Fatty acid binding protein |
| IpBrn01382 | K8 simple type II keratin |
| IpBrn01411 | NADH dehydrogenase subunit 5 and subunit 6 genes |
| IpBrn01425 | Tyrosine 3 monooxygenase/tryptophan 5-monooxygenase activation protein, theta polypeptide |
| IpBrn01443 | 40S ribosomal protein S27-1 mrna, complete cds |
| IpBrn02110 | 40S ribosomal protein S28 mrna, complete cds |
| IpHdk00050 | Translation elongation factor 1 alpha |
| IpHdk00054 | Ornithine decarboxylase antizyme |
| IpHdk00079 | LIM and SH3 domain-containing protein Lasp-1 |
| IpHdk00084 | 40S ribosomal protein S8 mrna, complete cds |
| IpHdk00103 | Alpha-tubulin |
| IpHdk00105 | Ribosomal protein L21 mrna, complete cds |
| IpHdk00106 | Y box protein 1 |
| IpHdk00141 | Dual specificity phosphatase |
| IpHdk00198 | Chaperonin cct6 |
| IpHdk00201 | Gelatinase B |
| IpHdk00285 | Ribosomal protein L36a mrna, complete cds |
| IpHdk00287 | Ribosomal protein L18 mrna, complete cds |
| IpHdk00290 | Receptor for activated protein kinase C |
| IpHdk00297 | 40S ribosomal protein S3 mrna, complete cds |
| IpHdk00299 | Ribosomal protein L27 mrna, complete cds |
| IpHdk00300 | Transaldolase |
| IpHdk00327 | Cytochrome b |
| IpHdk00337 | Ferritin heavy subunit |
| IpHdk00356 | Cytochrome c oxidase II |
| IpHdk00357 | NADH dehydrogenase subunit 2 |
| IpHdk00359 | *I. punctatus* MHC class I alpha chain icpu-uaa |
| IpHdk00365 | *I. punctatus* MHC class I alpha chain icpu-uca |
| IpHdk00494 | 40S ribosomal protein Sa mrna, complete cds |
| IpHdk00495 | 40S ribosomal protein S2 mrna, complete cds |
| IpHdk00625 | Ribosomal protein L4 mrna, complete cds |
| IpHdk00645 | Ribosomal protein L7 mrna, partial cds |
| IpHdk00683 | Cytochrome c oxidase III |
| IpHdk00721 | Cctd mrna for chaperonin containing TCP-1 delta |
| IpHdk00915 | *Lysozyme G (1,4-beta-n-acetylmuramidase)* |
| IpHdk01003 | Ribosomal protein L40 mrna, complete cds |
| IpHdk01008 | Ribosomal protein L8 mrna, complete cds |
| IpHdk01011 | Ribosomal protein L38 mrna, complete cds |
| IpHdk01014 | Ribosomal protein L22 mrna, complete cds |
| IpHdk01020 | Ribosomal protein L41 mrna, complete cds |
| IpHdk01029 | Atpase subunit 8 (atpase8) and atpase subunit 6 |
| IpHdk01038 | Ribosomal protein L17 mrna, complete cds |
| IpHdk01050 | Ribosomal protein L14 mrna, complete cds |
| IpHdk01056 | Ribosomal protein L27a mrna, complete cds |
| IpHdk01062 | Ribosomal protein L12 mrna, complete cds |
| IpHdk01077 | Ribosomal protein P2 mrna, complete cds |
| IpHdk01092 | Immunoglobulin light chain F class (igl) |
| IpHdk01122 | Ribosomal protein L31 mrna, complete cds |
| IpHdk01133 | Aldolase C |
| IpHdk01136 | Ribosomal protein L15 mrna, complete cds |
| IpHdk01138 | 40S ribosomal protein S11 mrna, complete cds |
| IpHdk01172 | Ribosomal protein L3 mrna, complete cds |
| IpHdk01186 | Ribosomal protein L32 mrna, complete cds |
| IpHdk01188 | Translation elongation factor 2 |
| IpHdk01201 | Ribosomal protein P0 mrna, complete cds |
| IpHdk01204 | NADH ubiquinone oxidoreductase subunit 4L |
| IpHdk01205 | Ribosomal protein L10 mrna, complete cds |
| IpHdk01210 | 40S ribosomal protein S10 mrna, complete cds |
| IpHdk01215 | Ribosomal protein L26 mrna, complete cds |
| IpHdk01225 | Cyclophilin D |
| IpHdk01254 | Danio rerio t-complex (chaperonins) polypeptide 1 (TCP1) |
| IpHdk01274 | 40S ribosomal protein S12 mrna, complete cds |
| IpHdk01279 | 40S ribosomal protein S4 mrna, complete cds |
| IpHdk01284 | Prepronerve growth factor homolog |
| IpHdk01290 | Ribosomal protein L29 mrna, complete cds |
| IpHdk01295 | Collagenase 3 precursor (matrix metalloproteinase-13) |
| IpHdk01305 | Ribosomal protein L13 mrna, complete cds |
| IpHdk01355 | *I. punctatus* clone icpu-a-2 mhc class ii antigen |
| IpHdk01435 | Ribosomal protein L9 mrna, complete cds |
| IpHdk01446 | Ribosomal protein L19 mrna, complete cds |
| IpHdk01450 | 40S ribosomal protein S6 mrna, complete cds |
| IpHdk01455 | ADP/ATP translocase |
| IpHdk01458 | Ribosomal protein L6 mrna, partial cds |
| IpHdk01497 | *I. punctatus* clone pg27 class g igl chain , vjc region |
| IpHdk01505 | Ribosomal protein L10a mrna, complete cds |
| IpHdk01506 | 14-3-3 protein beta |
| IpHdk01508 | Plastin 1 (I isoform) |
| IpHdk01514 | Beta-actin |
| IpHdk01515 | S-adenosylmethionine synthetase |
| IpHdk01558 | Ig rearranged H-chain mrna V-C-region |
| IpHdk01562 | Ribosomal protein L28 mrna, complete cds |
| IpHdk01592 | Ras-related C3 botulinum toxin substrate 2 |
| IpHdk01616 | Ribosomal protein L7a mrna, complete cds |
| IpHdk01625 | Ribosomal protein L37a mrna, complete cds |
| IpHdk01661 | Aminolevulinate synthase erythroid specific isoform |
| IpHdk01687 | 18S small subunit ribosomal RNA |
| IpHdk01777 | Ribosomal protein L5b mrna, complete cds |
| IpHdk01801 | Invariant chain-like protein 2 |
| IpHdk01904 | Human hypothetical protein HSPC014 |
| IpHdk01925 | Beta tubulin |
| IpHdk01934 | Ribosomal protein L36 mrna, complete cds |
| IpHdk01968 | 28s ribosomal RNA |
| IpHdk01994 | High mobility group protein 17 |
| IpHdk02015 | 40S ribosomal protein S3a mrna, partial cds |
| IpHdk02077 | Ribosomal protein P1 mrna, complete cds |
| IpHdk02148 | 40S ribosomal protein S17 mrna, complete cds |
| IpHdk02218 | Alpha-globin |
| IpHdk02262 | Trna-Thr |
| IpHdk02538 | Ribosomal protein L35a mrna, complete cds |
| IpHdk02547 | Danio rerio odorant receptor gene cluster |
| IpHdk02550 | Glutathione S-transferase |
| IpHdk02602 | Cofilin (small actin binding protein) 1, non-muscle |
| IpHdk02655 | Ribosomal protein L30 mrna, complete cds |
| IpHdk02672 | Kinesin light chain |
| IpHdk03011 | Ribosomal protein L5a mrna, complete cds |
| IpHdk03163 | 40S ribosomal protein S27a mrna, complete cds |
| IpHdk03186 | 40S ribosomal protein S13 mrna, complete cds |
| IpLvr00007 | 14 kda Apolipoprotein |
| IpLvr00023 | Intelectin |
| IpLvr00024 | Hemopexin-like protein |
| IpLvr00050 | Fibrinogen gamma |
| IpLvr00071 | Translocon-associated protein delta |
| IpLvr00097 | Alpha-2-HS glycoprotein |
| IpLvr00110 | Apolipoprotein A-I |
| IpLvr00118 | Apolipoprotein C-I |
| IpLvr00133 | Extracellular glutathione peroxidase |
| IpLvr00156 | Complement component C1R |
| IpLvr00162 | Fructose-1,6-bisphosphatase |
| IpLvr00286 | Liver-basic fatty acid binding protein |
| IpLvr00401 | Triglyceride lipase |
| IPLvr00507 | Trypsin IA |
| IPLvr00692 | Tryptophan dioxygenase |
| IpLvr00846 | Fibrinogen beta |
| IpLvr00910 | Alpha 1-microglobulin/bikunin |
| IpLvr00921 | Cysteine dioxygenase |
| IpLvr01006 | Transcobalamin |
| IpLvr01122 | Complement component C4B |
| IpLvr01239 | Alcohol dehydrogenase |
| IpLvr01445 | Direct IAP binding protein with low PI |
| IpLvr01473 | Complement B |
| IpLvr01654 | Heparin cofactor II |
| IpLvr01701 | Fibrinogen alpha |
| IpLvr02230 | Leukocyte cell-derived chemotaxin 2 |
| IpSkn00024 | Myosin light chain 2 |
| IpSkn00068 | 40S ribosomal protein S21 mrna, complete cds |
| IpSkn00226 | 40S ribosomal protein S24 mrna, complete cds |
| IpSkn00374 | Collagen, alpha 2 type I |
| IpSkn00392 | 40S ribosomal protein S15 mrna, complete cds |
| IpSkn00600 | 40S ribosomal protein S5 mrna, complete cds |
| IpSkn00604 | RAB11B, member RAS oncogene family |
| IpSkn00663 | 40S ribosomal protein S26-1 mrna, complete cds |
| IpSkn00749 | Troponin I |
| IpSkn00945 | 40S ribosomal protein S14 mrna, complete cds |
| IpSkn01028 | 40S ribosomal protein S27-2 mrna, complete cds |
| IpSkn01055 | 40S ribosomal protein S25 mrna, complete cds |
| IpSkn01243 | Parvalbumin |
| IpSkn01341 | Myosin light chain 3 |
| IpSkn01436 | 40S ribosomal protein S20 mrna, complete cds |
| IpSkn01450 | Keratin type I |
| IpSkn01504 | 40S ribosomal protein S29 mrna, complete cds |
| IpSkn01509 | Ubiquitin specific protease 9 |
| IpSkn01539 | SPARC, acidic calcium-binding glycoprotein |
| IpSkn01651 | Annexin max3 |
| IpSkn01753 | 40S ribosomal protein S26-2 mrna, complete cds |
| IpSkn01931 | 40S ribosomal protein S7 mrna, complete cds |
| IpSkn01936 | 40S ribosomal protein S16 mrna, complete cds |
| IpSkn01945 | Ribosomal protein L11 mrna, complete cds |
| IpSkn02068 | Keratin type II |
| IpSkn02205 | 40S ribosomal protein S18 mrna, complete cds |
| IpSkn02454 | Ribosomal protein L23 mrna, complete cds |
| IpSpn00012 | Hemoglobin beta chain |
| IpSpn00076 | Unknown |
| IpSpn00120 | Immunoglobulin heavy chain |
| IpSpn00138 | Ribosomal protein L18a mrna, complete cds |
| IpSpn00199 | Thymosin beta |
| IpSpn00218 | C1q related factor |
| IpSpn00265 | Unknown |
| IPSpn00295 | Cystatin E |
| IpSpn00363 | Interferon-induced protein 1-8D |
| IpSpn00408 | Ribosomal protein L37 mrna, complete cds |
| IpSpn00409 | Leukocyte DNA binding receptor |
| IpSpn00470 | MHC class II beta chain |
| IpSpn00474 | Unknown |
| IPSpn00667 | Interleukin-8 variant 3 |
| IpSpn00671 | Beta-2 microglobulin precursor |
| IpSpn00703 | Cysteine-rich protein 1 |
| IpSpn00983 | Galectin like protein |
| IpSpn01206 | Ribosomal protein L24 mrna, complete cds |
| IPSpn01216 | Invariant chain-like protein 1 |
| IpSpn01230 | Transposase |
| IpSpn01520 | Ribosomal protein L34 mrna, complete cds |
| IpSpn01530 | NADH dehydrogenase |
| IpSpn01550 | High affinity ige receptor gamma subunit |
| IpSpn01658 | Ubiquitin-like protein |
| IpSpn02021 | Ribosomal protein L35 mrna, complete cds |
| IpSpn02027 | Ribosomal protein L13a mrna, partial cds |

**Supplemental Table 2: Abundantly expressed transcripts (>2 copies/10,000 transcripts) in catfish EST collection in NCBI’s dbEST following the current project. Approximately 40,000 catfish ESTs were assembled. Indicated clones were used as drivers in subtraction of normalized cDNA libraries currently being sequenced by JGI.**

| **# of Seq in Contig** | **Clone Used for Subtraction** | **Identity** |
| --- | --- | --- |
| 216 | IpLvr00376 | Cytochrome c oxidase subunit 1 |
| 180 | IpHdk03132 | Beta actin |
| 178 | IpSkn01886 | Beta actin |
| 143 | AUF_IfSpn_236_G01 | NK-lysin |
| 137 | IpHdk03087 | Ribosomal protein S2 |
| 135 | IpHdk00735 | Elongation factor 1 alpha |
| 122 | IpHdk00356 | Cytochrome c oxidase II |
| 95 | IpLvr00727 | Apolipoprotein |
| 95 | IpHdk01864 | Beta actin |
| 91 | IpHdk00013 | Thymosin beta |
| 91 | IpLvr00132 | Hemoglobin beta chain |
| 88 | IpLvr00354 | Apolipoprotein A-I |
| 79 | IpSpn00982 | Ribosomal protein L11 |
| 79 | IpHdk01888 | Granzyme-like I |
| 78 | IpHdk00588 | Profilin 2 |
| 77 | IpSkn01697 | Ribosomal protein L41 |
| 75 | IpHdk01110 | Beta-globin |
| 75 | IpHdk01179 | Norvegicus laminin receptor 1 |
| 73 | IpSkn00968 | Ribosomal protein L6 |
| 73 | IpSkn01058 | Cytochrome b |
| 73 | IpSkn01274 | Ribosomal protein L38 |
| 72 | IpLvr00174 | Warm temperature acclimation-related 65 kda |
| 72 | IpSkn00724 | Creatine kinase |
| 71 | IpSpn01634 | Ribosomal protein l35a |
| 69 | IpHdk00959 | Ferritin heavy subunit |
| 68 | IpSkn01411 | Ribosomal protein S12 |
| 68 | IpSkn01410 | Cytochrome c oxidase subunit III |
| 68 | AUA_IpPit00312 | Proopiomelanocortin |
| 64 | IpSpn00797 | Elastase 3 precursor |
| 64 | IpSkn02361 | Ribosomal protein L32 |
| 62 | IpSkn01064 | Ribosomal protein l37a |
| 61 | IpSkn02248 | Ribosomal protein S20 |
| 61 | IpSpn00016 | Ribosomal protein large P2 |
| 61 | IpHdk01427 | Acidic ribosomal phophoprotein P0 |
| 61 | IpHdk01509 | Trna-Val gene |
| 59 | IpSkn01978 | Ribosomal protein L35 |
| 57 | IpSkn00017 | Ribosomal protein L30 |
| 56 | IpSkn01893 | Beta-2 microglobulin precursor |
| 56 | IpHdk00436 | Ribosomal protein L24 |
| 56 | IpSkn01938 | Ribosomal protein l13a |
| 55 | AUB_IfLvr00158 | Trypsinogen |
| 55 | IpHdk01779 | Ictacalcin |
| 53 | IpHdk01990 | Ribosomal protein S9 |
| 52 | AUB_IfLvr00084 | Trypsin III precursor |
| 50 | IpHdk01523 | Ribosomal protein l7a |
| 50 | IpHdk01148 | Adenine nucleotide translocase |
| 50 | IpHdk02041 | Ribosomal protein S24 |
| 48 | IpSpn02011 | Glyceraldehyde-3-phosphate dehydrogenase |
| 47 | IpSpn00827 | Thymosin/interferon-inducible |
| 47 | IpSkn01569 | Ribosomal protein L12 |
| 47 | IpHdk00272 | Trna-Val gene |
| 46 | IpLvr01504 | Ribosomal protein S3 |
| 44 | IpHdk00705 | Ribosomal protein S4 |
| 44 | IpHdk01587 | BMS prohibitin |
| 43 | IpHdk00421 | Ribosomal protein L18 |
| 43 | IpSpn01719 | Ribosomal protein S14 |
| 41 | IpHdk00942 | Creatine kinase |
| 40 | IpHdk00400 | Ribosomal protein L3 |
| 39 | IpHdk02015 | Ribosomal protein s3a/v-Fos transformation effector |
| 38 | IpSpn00597 | Ribosomal protein S15 |
| 38 | IpSkn01686 | Ribosomal protein S18 |
| 38 | IpSkn00023 | Ribosomal protein L10 |
| 38 | IpBrn00076 | Ribosomal protein L10A |
| 38 | IpLvr01392 | Dihydroxyvitamin D3-induced protein |
| 37 | IpSpn00150 | Invariant chain-like protein 2 |
| 37 | IpHdk00708 | Elongation factor 2 |
| 36 | IpLvr01145 | Warm-temperature-acclimation-related protein |
| 36 | IpLvr01881 | Fibrinogen gamma A chain |
| 36 | IpLvr00469 | Ribosomal protein L9 |
| 35 | IpBrn00335 | Ribosomal protein L5b 60S |
| 35 | IpSpn00789 | Ribosomal protein L17 |
| 35 | IpHdk01100 | Smooth muscle myosin |
| 35 | IpHdk00049 | 18S small subunit ribosomal RNA gene |
| 34 | IpHdk02260 | Ribosomal protein L19 |
| 34 | IpHdk01166 | Ribosomal protein L4 |
| 34 | IpHdk02602 | Cofilin (small actin binding protein) |
| 33 | IpLvr02066 | Golgi 4-transmembrane spanning transporter MTP |
| 33 | IpLvr01466 | NADH dehydrogenase subunit 2 |
| 33 | IpSkn01837 | Ribosomal protein L36 |
| 33 | IpHdk02667 | High mobility group protein 17 |
| 32 | IpHdk01452 | Ribosomal protein L15 |
| 32 | IpSpn00144 | Unknown |
| 32 | IpBrn02081 | Ribosomal protein S8 |
| 32 | IpLvr00637 | Translationally-controlled tumor protein |
| 32 | IpSkn01109 | Unknown |
| 32 | IpHdk00012 | Heat shock protein 70 |
| 32 | IpHdk02165 | Y box protein 1 |
| 31 | IpHdk01414 | Unknown |
| 31 | IpHdk02615 | Coronin actin-binding protein pp66 |
| 31 | AUA_IpPit00260 | Growth hormone |
| 30 | IpHdk00290 | Activated protein kinase C |
| 30 | IpLvr00988 | Intelectin |
| 30 | IpLvr00201 | Trypsin IA |
| 30 | IpHdk02585 | Alpha-globin IV globin zeta |
| 30 | IpHdk00960 | Alpha-globin |
| 30 | IpHdk01877 | Ribosomal protein S11 |
| 30 | IpSkn00171 | Elongation factor 1-beta homolog |
| 29 | IpHdk00592 | Ribosomal protein L21 |
| 29 | IpHdk01710 | Ribosomal protein S10 |
| 29 | IpSpn01578 | Trypsin IA |
| 29 | AUA_IpInt00222 | Ribosomal protein L22 |
| 28 | IpHdk01556 | Beta-actin |
| 27 | AUA_IpTrk00031 | Unknown |
| 27 | AUF_IfLvr_214_P09 | Chymotrypsinogen B1 |
| 26 | IfHdk00925 | Beta-2 microglobulin precursor |
| 26 | IpHdk02304 | Cold inducible RNA binding protein |
| 26 | IpHdk03089 | Heat shock protein |
| 26 | IpSkn02034 | Ribosomal protein S29 |
| 26 | IfHdk01186 | Ribosomal protein L7 |
| 26 | IpLvr00002 | Microfibril-associated glycoprotein 4 |
| 26 | IpSkn00973 | Muscle-specific beta 1 integrin binding protein |
| 26 | IpLvr00359 | Apolipoprotein C-I |
| 26 | IpHdk01254 | T-complex (chaperonins) polypeptide 1 |
| 26 | IpHdk00574 | Unknown |
| 26 | IpHdk01420 | Alpha-tubulin |
| 25 | IpHdk02309 | Receptor for activated protein kinase C |
| 25 | IpSpn01183 | Pancreatic procarboxypeptidase B |
| 25 | IpLvr00684 | Ribosomal protein L31 |
| 25 | IpLvr01646 | Alpha-microglobulin/HI-30 precursor |
| 25 | IpSkn01848 | Cyclophilin D |
| 25 | IpSkn00011 | Putative oncoprotein nm23 |
| 25 | IpHdk00492 | SM22 alpha |
| 25 | IpLvr00102 | Serine protease inhibitor |
| 24 | IpBrn01359 | Ribosomal protein S27 |
| 24 | AUF_IfLvr_214_B07 | Complement component C3-4 |
| 24 | IpLvr02080 | NADH ubiquinone oxidoreductase subunit 4L |
| 24 | IpHdk00238 | Invariant chain-like protein |
| 24 | IpHdk00659 | Ribosomal protein l36a |
| 24 | IpBrn01279 | Ependymin (gd) precursor gene |
| 24 | IpSkn02578 | Cysteine-rich heart protein |
| 24 | IpHdk00106 | Y box protein 1 |
| 23 | IpHdk01188 | Translation elongation factor 2 |
| 23 | IpSpn01561 | Trypsin III |
| 23 | IpPitR00068 | Ribosomal phosphoprotein P1 |
| 23 | IpPitF00024 | Cytochrome c oxidase |
| 23 | IpSpn01490 | High affinity ige receptor gamma subunit |
| 23 | IpLvr02384 | Ribosomal protein L14 |
| 23 | IpSkn02576 | Ribosomal protein S16 |
| 23 | IpHdk00355 | Unknown |
| 22 | IpSkn01200 | Unknown |
| 22 | IpHdk00585 | Monoclonal non-specific suppressor factor beta |
| 22 | IpSpn00052 | Ubiquitously expressed (fox derived) |
| 22 | IpHdk00758 | Ribosomal protein L27 |
| 22 | IpBrn01512 | Ribosomal protein S27 |
| 22 | IpBrn01004 | Unknown |
| 22 | IpLvr01003 | Helicase II/Gu protein gene |
| 22 | IpHdk00415 | ATP synthase, H+ transporting mitochondrial F0 complex |
| 22 | IpHdk00609 | Unknown |
| 21 | IpSkn00946 | PAI-1 mrna-binding protein |
| 21 | IpLvr00211 | Fibrinogen gamma |
| 21 | No our clone | Unknown |
| 21 | IpHdk00214 | Nascent-polypeptide-associat |
| 21 | IpHdk01959 | B-tubulin |
| 21 | IpSkn00196 | Predicted RNA-binding protein homologous to eukaryotic snrp |
| 21 | IpSkn00068 | Ribosomal protein S21 |
| 21 | IpLvr00068 | Poly(A)-binding protein, cytoplasmic 4 |
| 21 | AUF_IpSpn_63_b17 | Carboxypeptidase A1 |
| 21 | IpSpn01565 | Interleukin-8 |
| 21 | IpSkn00906 | 16S rrna |
| 20 | IpSpn00181 | Prothymosin, alpha |
| 20 | IpHdk02590 | Ribosomal protein S23 |
| 20 | IpSkn00378 | Ribosomal protein S8 |
| 20 | IpLvr00445 | Ribosomal protein S30 |
| 20 | IpBrn00176 | Cytochrome c oxidase polypeptide VIC |
| 20 | IpHdk01098 | ARP2/3 protein complex (regulator of actin polymerization) subunit p41-Arc (ARC41) |
| 20 | IpHdk00773 | Ribosomal protein l18a |
| 20 | IpHdk01178 | Translation initiation factor SUI1 |
| 19 | IpHdk01352 | MHC class I alpha chain |
| 19 | IpSkn02560 | Ribosomal protein S11 |
| 19 | IpSpn01525 | Ubiquitin |
| 19 | IpHdk02673 | Ribosomal protein S6 |
| 19 | IpHdk00146 | Ribosomal protein L5 |
| 19 | IpLvr01501 | Alcohol dehydrogenase 1 |
| 18 | IpSpn00267 | Beta-2 microglobulin precursor |
| 18 | IpLvr01885 | MHC class I alpha chain Icpu-UBA |
| 18 | IpSpn00102 | Ribosomal protein L23 |
| 18 | IpSkn00185 | Myosin light chain 2 |
| 18 | IpHdk01968 | 28s ribosomal rna |
| 18 | IpSkn00005 | Elongation factor 1 gamma |
| 18 | IpSpn00329 | Ribosomal protein S17 |
| 18 | IpHdk01029 | Atpase subunit 8 (atpase8) and atpase subunit 6 |
| 18 | IpSkn00328 | Cd63 antigen |
| 18 | IpSpn00783 | 14-3-3 protein beta |
| 18 | AUF_IpSpn_69_k09 | Ferritin-H subunit |
| 18 | IpSkn00252 | Ribosomal protein S5 |
| 17 | IpSkn00957 | Ribosomal protein L22 |
| 17 | IpHdk02020 | Ribosomal protein S25 |
| 17 | IpBrn01154 | Translation initiation factor 1A |
| 17 | IpLvr01848 | Predicted O-methyltransferase |
| 17 | IpHdk01592 | Ras-related C3 botulinum toxin substrate 2 |
| 17 | IpHdk03046 | Alpha-tubulin |
| 17 | IpHdk02702 | Beta actin |
| 16 | IpHdk01507 | Arp2/3 protein complex (regulator of actin polymerization) subunit p20 (ARC20) |
| 16 | IpHdk01862 | Ribosomal S13 protein |
| 16 | IpHdk00441 | Ribosomal protein L8 |
| 16 | IpHdk02706 | Growth factor receptor-bound protein 2 |
| 16 | IpHdk03054 | Ras-related nuclear protein Ran |
| 16 | IpHdk03195 | Lactate dehydrogenase-A (Ldh-A) |
| 15 | IpLvr01717 | Alpha-2-hs-glycoprotein |
| 15 | IpHdk02674 | Lancl1 protein |
| 15 | IpLvr01700 | Triosephosphate isomerase(D-glyceraldehyde 3-phosphate ketol-isomerase) |
| 15 | IpSpn01727 | MDV1 S2 (Marek's disease virus serotype 1) |
| 15 | IpLvr00846 | Fibrinogen beta |
| 15 | IpBrn00598 | Ferritin middle subunit |
| 15 | IpSkn02356 | Translation initiation factor 5A |
| 15 | IpHdk00533 | Ribosomal protein l27a |
| 15 | IpSpn01563 | MHC class II beta chain (icpudab*01 allele) |
| 15 | IpBrn00820 | L-plastin |
| 14 | IpHdk00381 | Ribosomal protein L31 |
| 14 | IpHdkF00365 | MHC class I alpha chain Icpu-UCA |
| 14 | IpSkn01352 | Ribosomal protein L39 |
| 14 | IpBrn01426 | Fatty acid binding protein |
| 14 | IpLvr00610 | Cytochrome c oxidase polypeptide VB (VI) |
| 14 | AUA_IpSto00164 | Ribosomal protein L30 |
| 14 | IpHdk01986 | Natural killer cell enhancement factor |
| 14 | IpSpn01241 | Eukaryotic translation initiation factor 3, subunit 5 (epsilon, 47kd) (EIF3S5) |
| 14 | IpLvr01901 | Liver-basic fatty acid binding protein |
| 14 | IpSkn00583 | Urokinase receptor |
| 14 | IpHdk01726 | Heat shock protein hsp90beta |
| 14 | IpSkn00163 | Ribosomal protein L41 |
| 14 | IpHdkF00331 | Ribosomal protein S19 |
| 14 | IpHdk02549 | NADH dehydrogenase subunit 1 |
| 14 | IpHdk02001 | Lymphocyte cytosolic protein 1 (L-plastin) |
| 13 | IpLvr00194 | Alpha-1-microglobulin/bikunin |
| 13 | IpHdk03062 | Unknown |
| 13 | IpSkn01432 | NADH dehydrogenase subunit 3 |
| 13 | IpLvr00887 | NADH dehydrogenase subunit 4L |
| 13 | AUF_IpPit_32_c01 | Unknown--not in genbank |
| 13 | IpHdk03084 | H1d-histone and H2B-histone |
| 13 | IpSkn01619 | Translation initiation factor 3 subunit 3 |
| 13 | IpSkn00917 | Epididymal secretory protein E1 |
| 13 | IpHdk02557 | Lps-induced tnf-alpha factor |
| 13 | AUF_IpInt_53_j20 | S-adenosylhomocysteine hydrolase |
| 13 | IpLvr00702 | Testis enhanced gene transcript-like protein |
| 13 | AUA_IpSto00004 | F-actin capping protein alpha-1 subunit |
| 13 | IpHdk00057 | Unknown |
| 13 | IpSpn00860 | Unknown |
| 13 | IpHdk02562 | Unknown |
| 13 | AUF_IpOva_18_c05 | Unknown |
| 13 | AUF_IpHdk_44_p18 | Activating transcription factor 4 |
| 12 | AUF_IfLvr_215_h23 | Unknown |
| 12 | AUF_IfLvr_222_f16 | Warm-temperature-acclimation-related-65 |
| 12 | IpLvr02301 | Wap65 (Warm temperature acclimation-related 65 kda protein, Hemopexin-like) |
| 12 | AUF_IpSpn_63_g04 | Chimeric AFGP/trypsinogen-like serine protease precursor |
| 12 | IpSkn00663 | Ribosomal protein S26 |
| 12 | IpHdk02628 | Calmodulin |
| 12 | IpHdk02161 | ARP2/3 complex (regulator of actin polymerization) subunit p21- Arc |
| 12 | IpHdk00054 | Ornithine decarboxylase antizyme |
| 12 | IpLvr01108 | Elastase |
| 12 | IpLvr00962 | Ribosomal protein L13 |
| 12 | IpBrn02110 | Ribosomal protein S28 |
| 12 | IpLvr01141 | Complement component C4B |
| 12 | IpLvr01701 | Fibrinogen alpha subunit |
| 12 | IpSpn00296 | Ribosomal protein L34 |
| 12 | AUF_IpHdk_45_d18 | Grp58-prov protein |
| 12 | IpHdk00410 | Ribosomal protein L28 |
| 12 | IpBrn01321 | Ribosomal protein L28 |
| 12 | AUF_IpHdk_41_e10 | Serine protease TADG15 |
| 12 | IpSkn01923 | Ribosomal protein L41 |
| 12 | IpHdk00122 | Ribosomal protein L41 |
| 12 | IpSpn00435 | Mitochondrial phosphate carrier protein precursor |
| 11 | IpLvr00071 | Translocon-associated protein delta |
| 11 | IpSpn00239 | Cold inducible RNA-binding protein alpha |
| 11 | IpSkn01048 | Glyceraldehyde-3-phosphate dehydrogenase |
| 11 | IpSkn01486 | Human TB2 gene |
| 11 | IpHdk02631 | Testis-specific alpha-tubulin |
| 11 | IpSpn00819 | Calmodulin |
| 11 | AUF_IpSpn_68_o24 | Chaperonin-containing TCP-1 complex beta chain |
| 11 | IpBrn00392 | Unknown |
| 11 | IpSkn00679 | Sorcin |
| 11 | IpHdk01612 | Translation initiation factor eif4a I |
| 11 | IpSpn02036 | Deleted in split-hand/split-foot 1 region (DSS1) |
| 11 | IpHdk03187 | Arp2/3 protein complex (regulator of actin polymerization) subunit p20 (ARC20) |
| 11 | IpSkn02051 | Chandra protein |
| 11 | IpLvr02303 | Alpha2-HS glycoprotein |
| 11 | AUF_IpSpn_67_m19 | Amylase-3 |
| 11 | IpLvr00245 | Apolipoprotein B |
| 11 | IpSkn01035 | Unknown |
| 11 | AUF_IpInt_58_b13 | HIF-1 responsive RTP801 |
| 11 | AUA_IpPit00153 | Proopiomelanocortin |
| 10 | AUF_IfLvr_214_k16 | Unknown |
| 10 | AUF_IfLvr_213_h07 | Unknown |
| 10 | IpHdk02024 | Unknown |
| 10 | AUF_IpSto_13_g03 | Peptidylprolyl isomerase A |
| 10 | IpSkn01390 | Ribosomal protein S2 |
| 10 | AUF_IpSto_10_m24 | CDK105 protein |
| 10 | IfHdk00427 | Phosphatidylinositol 3-kinase p45 subunit |
| 10 | IpSpn00360 | ATP synthase, oligomycin sensitivity conferring protein |
| 10 | IpHdk02659 | Phosphoglycerate mutase type B subunit |
| 10 | AUF_IpHdk_41_d18 | Purine nucleoside phosphorylase |
| 10 | IpHdk03057 | Unknown |
| 10 | IpHdk02637 | Transaldolase |
| 10 | IpSpn00964 | 15 kda selenoprotein |
| 10 | IpLvr00632 | Lysosome-associated membrane glycoprotein 1 |
| 10 | IpLvr00596 | Inhibitor of apoptosis protein (IAP |
| 10 | IpSpn00162 | Unknown |
| 10 | IpHdk01559 | Cathepsin S gene, exon 3 |
| 10 | AUF_IpHdk_44_b08 | Unknown |
| 10 | IpBrn01605 | Unknown |
| 10 | AUF_IpOva_20_a11 | Acetyl-Coenzyme A dehydrogenase |
| 10 | AUB_IfHrt_203_G13 | Tubulin, alpha 1 |
| 10 | IpLvr00553 | Melanotransferrin |
| 10 | AUF_IfInt_206_f02 | Unknown |
| 10 | IpHdk02191 | Similar to Beta-actin |
| 10 | IpHdk00430 | Ribosomal protein l9 |
| 9 | IpSpn00457 | Unknown |
| 9 | IpHdk01018 | Dendritic cell protein (GA17 protein) |
| 9 | AUF_IfLvr_212_p03 | Unknown |
| 9 | IpLvr00133 | Extracellular glutathione peroxidase |
| 9 | IpLvr01232 | Cofilin 2 |
| 9 | IpSpn01480 | Ribosomal protein L26 |
| 9 | IfHdk00538 | Elastase 4 precursor |
| 9 | IpHdk02233 | Cctd mrna for chaperonin containing TCP-1 delta |
| 9 | IpHdk00622 | Rap1b |
| 9 | IpHdk03004 | Microsomal signal peptidase (18 kd) |
| 9 | AUF_IpOva_21_p12 | Cytosolic malate dehydrogenase thermostable form |
| 9 | IpLvr01026 | Malate dehydrogenase |
| 9 | IpLvr02170 | Junb transcription factor |
| 9 | IpHdk00776 | Unknown |
| 9 | AUA_IpOva00109 | Hypoxia induced gene 1 (HIG1) |
| 9 | IpLvr00683 | Phosphoglycerate kinase |
| 9 | IpBrn00550 | Unknown |
| 9 | IpLvr02002 | CCCH zinc finger protein C3H-2 |
| 9 | IpSkn02528 | Translation initiation factor 3 subunit 6 |
| 9 | IpSkn00874 | Trna-Thr, trna-Pro, D-loop; and trna-Phe genes |
| 9 | IpBrn01021 | Unknown |
| 9 | IpLvr00969 | Nephrosin |
| 9 | AUA_IpTes00367 | Unknown |
| 9 | IpBrn01171 | Apolipoprotein E |
| 9 | IpSkn01021 | Human SH3 domain binding glutamic acid-rich protein like |
| 9 | IpLvr00018 | Fetuin-like protein IRL685 |
| 9 | IpHdk00700 | Unknown |
| 9 | IpLvr01894 | Unknown |
| 9 | IpLvr01729 | Polyubiquitin |
| 9 | IpSpn00247 | ATP synthase beta-subunit |
| 9 | IpHdk00199 | Icpu-A-2 MHC class II antigen mrna |
| 9 | IpSkn01016 | Ribosomal protein l23a |
| 9 | IpSkn00850 | Ribosomal protein L38 |
| 9 | IfHdk00523 | Unknown |
| 9 | IpHdk00647 | Proliferating cell nuclear antigen |
| 9 | IpSkn01873 | Unknown |
| 9 | IpSpn01619 | 18S small subunit ribosomal RNA gene |
| 9 | IfHdk00747 | Cytochrome c oxidase polypeptide i |
| 9 | AUF_IpSto_10_ p01 | Unknown |
| 9 | IpSkn01320 | Ribosomal protein L29 |
| 8 | IpHdk00956 | Unknown |
| 8 | IpSkn02251 | MHC class I alpha chain |
| 8 | IpPitF00097 | Growth hormone |
| 8 | IpHdk02687 | H-Fatty acid binding protein |
| 8 | AUA_IpPit00238 | Atpase, H+ transporting, lysosomal |
| 8 | IpSpn00520 | Danio rerio helix-loop-helix protein (Id6) |
| 8 | IpHdk01135 | Alpha enolase |
| 8 | IpLvr00072 | Kininogen I |
| 8 | IpLvr01353 | Adenine nucleotide Translocase |
| 8 | AUF_IpHdk_41_m05 | RAN binding protein 1 |
| 8 | AUF_IpOva_19_k05 | Selenoprotein M precursor |
| 8 | IpHdk01454 | Calcium-dependent protease, small subunit |
| 8 | AUF_IpSpn_61_d12 | Translation initiation factor 2 |
| 8 | IpSkn01046 | Translation initiation factor 5A |
| 8 | IpHdk01432 | Unknown |
| 8 | IpLvr01154 | Unknown |
| 8 | IpHdk00079 | LIM and SH3 domain-containing protein Lasp-1 |
| 8 | AUF_IpTrk_25_e21 | Serine/threonine protein kinase 17A+B1256 |
| 8 | IpSpn00281 | Ribosome associated membrane protein RAMP4 |
| 8 | IpSkn01203 | Human HSPC280 |
| 8 | IpHdk01656 | Proteasome activator subunit 2 |
| 8 | IpHdk01046 | Proteasome activator subunit 1 |
| 8 | IpHdk01164 | Cathepsin L |
| 8 | IpLvr00162 | Fructose-1,6-bisphosphatase 2 |
| 8 | AUF_IpGil_05_i09 | Microtubule-associated protein, RP/EB family, member 1 |
| 8 | IpHdk02223 | Unknown |
| 8 | AUA_IpMsl00288 | Proteasome subunit beta type 3 |
| 8 | IpLvr00299 | Mannose-binding protein-associated serine protease |
| 8 | IpLvr01836 | Transcobalamin |
| 8 | IpHdk02267 | 20S proteasome subunit C2 |
| 8 | AUF_IpSto_11_n05 | Unknown |
| 8 | IpBrn00872 | Sat-prov protein |
| 8 | IpHdk02688 | High mobility group protein 2 |
| 8 | IpHdk03051 | Putative 26S protease subunit |
| 8 | IpLvr00555 | Retinol dehydrogenase |
| 8 | IpLvr01184 | Beta glutamine synthetase |
| 8 | IpLvr01707 | Unknown |
| 8 | AUF_IfLvr_220_i24 | Chain a, bovine mitochondrial f1-atpase |
| 8 | AUF_IpTes_24_f21 | Unknown |
| 8 | IpHdk01513 | Adenylyl cyclase-associated protein |
| 8 | IpSpn00768 | Unknown |
| 8 | IpLvr01077 | Loc402873 |
| 8 | IpSkn01926 | Mcl-1a |
| 8 | IpSkn01915 | Rho GDI, GDP dissociation inhibitor, |
| 8 | IpLvr02306 | Unknown |
| 8 | AUF_IpSpn_66_j10 | Beta thymosin |
| 8 | AUF_IpSto_10_f10 | Unknown |
| 8 | IpHdk02694 | Uncoupling protein 2 |
| 8 | AUF_IfLvr_212_g19 | Heat shock cognate 71 kda |
| 8 | AUF_IpSpn_64_o05 | Unknown |
| 8 | AUF_IpSpn_62_i21 | Unknown |
| 8 | IpSkn00791 | Ribosomal protein S19 |
